# Distracting the Mind Improves Performance: An ERP Study

**DOI:** 10.1371/journal.pone.0015024

**Published:** 2010-11-24

**Authors:** Stefan M. Wierda, Hedderik van Rijn, Niels A. Taatgen, Sander Martens

**Affiliations:** 1 Neuroimaging Center, University of Groningen, Groningen, The Netherlands; 2 Department of Neuroscience, University Medical Center Groningen, Groningen, The Netherlands; 3 Department of Psychology, University of Groningen, Groningen, The Netherlands; 4 Department of Artificial Intelligence, University of Groningen, Groningen, The Netherlands; Kyushu University, Japan

## Abstract

**Background:**

When a second target (T2) is presented in close succession of a first target (T1), people often fail to identify T2, a phenomenon known as the attentional blink (AB). However, the AB can be reduced substantially when participants are distracted during the task, for instance by a concurrent task, without a cost for T1 performance. The goal of the current study was to investigate the electrophysiological correlates of this paradoxical effect.

**Methodology/Principal Findings:**

Participants successively performed three tasks, while EEG was recorded. The first task (standard AB) consisted of identifying two target letters in a sequential stream of distractor digits. The second task (grey dots task) was similar to the first task with the addition of an irrelevant grey dot moving in the periphery, concurrent with the central stimulus stream. The third task (red dot task) was similar to the second task, except that detection of an occasional brief color change in the moving grey dot was required. AB magnitude in the latter task was significantly smaller, whereas behavioral performance in the standard and grey dots tasks did not differ. Using mixed effects models, electrophysiological activity was compared during trials in the grey dots and red dot tasks that differed in task instruction but not in perceptual input. In the red dot task, both target-related parietal brain activity associated with working memory updating (P3) as well as distractor-related occipital activity was significantly reduced.

**Conclusions/Significance:**

The results support the idea that the AB might (at least partly) arise from an overinvestment of attentional resources or an overexertion of attentional control, which is reduced when a distracting secondary task is carried out. The present findings bring us a step closer in understanding why and how an AB occurs, and how these temporal restrictions in selective attention can be overcome.

## Introduction

Although the human mind is quite capable of performing multiple tasks at the same time, the multitasking brain does not always react accurate or fast enough in complex situations. An obvious example is that the likelihood of traffic accidents increases when driving is combined with the concurrent use of a cellular phone, especially when the level of complexity increases and additional attentional control is required [1]. However, under some circumstances described below, an increase in cognitive load can reduce temporal restrictions in attention, such that one's multitasking performance improves rather than deteriorates.

Within the lab, restrictions in temporal attention are for instance revealed when two targets (e.g., letters) are presented in close temporal proximity within a sequential stream of distractor stimuli (e.g., digits). When the second target (T2) is presented within ∼200 to 500 ms after the onset of the first target (T1), participants often fail to report the second target, reflecting the occurrence of an attentional blink (AB) [2]. It has been shown, however, that identification performance can increase when a second task is added to this so-called AB task [3]–[6]. The aim of the current study was to investigate this paradoxical phenomenon in more detail.

For two decades, the AB has been a major topic in attention research because it is informative about the rate at which stimuli can be encoded into consciously accessible representations. As the AB can be obtained using a variety of stimuli and task conditions, it is thought to reflect a very general property of perceptual awareness (for a review, see [7]).

However, recent findings suggest that the AB is unlikely to reflect a hard-wired bottleneck. For instance, the presence of perceptual, spatial, or temporal cues has been found to reduce the magnitude of the AB (defined as the percentage of decrement in T2 performance within the AB period relative to T1 performance), presumably by redistribution or accelerated allocation of attention (e.g., [8]–[10]). As mentioned above, it has also been found that adding a source of distraction during the AB task can attenuate the AB. In an experiment by Olivers and Nieuwenhuis [4], participants had to do an AB task in four different conditions. In one condition, participants did a standard AB task. In the other three conditions participants were financially rewarded for their performance, do free association (e.g., think about their most recent holiday) or listen to music during the AB task. In the latter two conditions, the AB was substantially reduced, while rewarding the participants did not make any difference. Although the initial finding could not be fully replicated [5], others have shown that task-irrelevant visual motion or flicker [3], a change in task instruction [11], or even a concurrent secondary task [6] can attenuate the AB as well.

In the study of Taatgen et al. [6], participants had to perform an AB task together with a concurrent secondary task that required participants to detect the occurrence of a red dot. The AB task consisted of identifying target letters amongst distractor digits. The detection task contained a grey dot presented in peripheral vision that moved in a circular direction concurrent to the stimulus stream of the AB task. In 25% of the trials, the grey dot turned red for a brief moment. At the end of a trial, participants were instructed to report whether or not a red dot occurred during the trial. Trials in which the red dot actually turned red were excluded from analyses, as the red dot itself induced an AB. AB magnitude was found to be smaller in the concurrent task condition than in a control condition without dots.

Supported by computer simulations, Taatgen et al. [6] argued that an AB might be caused by an overexertion of cognitive control, which is suspended when a secondary task is concurrently performed during the AB task. In addition, their computational model provided a first explicit account of target selection processes in individuals who do not show an AB, referred to as ‘non-blinkers’ [12].

Whereas individual differences in AB research are usually ignored, Martens, Munneke, Smid, and Johnson [12] demonstrated the existence of large individual differences in the magnitude of the AB, with some individuals showing little or no AB whatsoever (‘non-blinkers’). Measuring event-related potentials (ERP), they found significant electrophysiological differences between non-blinkers and individuals who do show a strong AB (‘blinkers’). More specifically, non-blinkers showed earlier target-related parietal activity (reflected in the P3, a component associated with the updating of working memory). In addition, they exhibited more target-related frontal activity (reflected in the FSP, a component associated with early target selection processes), as well as reduced distractor-related frontal activity.

Martens, Elmallah, London, and Johnson [13] correlated the magnitude of the AB with the size of the P3 amplitude. In their experiment, the frequency of the first target in a standard AB paradigm was manipulated. Whenever an infrequent first target was presented, the P3 amplitude evoked by T1 and the AB magnitude increased. Martens and colleagues suggested that the amplitude of the P3 of T1 reflects the amount of attention or resources allocated to consolidate T1 [13]–[16] and that the more resources allocated to T1, the smaller the chance for T2 to receive sufficient attention, reflected in the occurrence of an AB.

According to Taatgen and colleagues [6], most individuals tend to protect memory consolidation of a first target by temporarily blocking the detection of subsequent targets, resulting in an AB. They suggested that non-blinkers refrain from exerting this protective control, thus avoiding the AB. This notion could explain the paradoxical finding of improved performance during dual-tasking: When blinkers are given a secondary task during the AB, the brain might be taxed to such an extent that the exertion of protective control is relaxed, resulting in an attenuated AB (also see [5]).

An intriguing question is whether these ‘induced non-blinkers’ (i.e. individuals showing a reduced AB magnitude due to distraction manipulations) adopt a similar processing strategy as the natural non-blinkers reported by Martens and colleagues [12]. If so, induced non-blinkers should show similar patterns of frontal and parietal brain activity as previously observed in natural non-blinkers. That is, a shift in target-related P3 latencies would be expected, along with a larger target-related FSP component, as well as reduced distractor-related activity [12]. In addition, because a lower P3 amplitude of the first target is associated with a smaller AB magnitude, one would expect the P3 amplitude to be lower in the concurrent tasks condition than in the standard AB task condition.

However, different patterns of brain activity might be expected if natural and induced non-blinkers are qualitatively different in the way they perform the AB task. The aim of the current study was to test this and to reveal what influence the addition of a secondary task to a standard AB task has on the various components in the ERP. To that end, we adapted the task used by Taatgen et al. [6] to include event-related potential (ERP) recordings, and investigated the FSP, P3, and overall distractor-related activity. Contrary to Taatgen et al., we compared performance on trials in the experimental condition with that of trials in an additional control condition that featured identical input and only differed in task instructions, allowing a direct comparison of behavioral as well as ERP data.

## Methods

### Participants

Thirty psychology students from the University of Groningen were recruited via an online sign-up program. They received course credits for their participation in the experiment. The Neuroimaging Center Institutional Review Board and the Ethics Committee of Psychology at the university of Groningen approved the experimental protocol. Written informed consent was obtained prior to the experiment. Five participants were excluded from the analyses due to bad performance (i.e. they had an accuracy <50% on the first target). Another participant was excluded from the analyses due to artifacts in more than 50% of the EEG data segments. The remaining twenty-four participants were aged 18 to 33 (mean = 21.25) with normal or correct-to-normal visual acuity.

### Stimuli and Apparatus

The software package E-Prime was used to generate stimuli and to collect responses, running under Windows XP on a PC with a 17-inch monitor. Stimuli consisted of consonants (excluding “Q”, “V”, and “Y”) and digits (excluding “0” and “1”) and were presented in black on a white background in a bold 12-point Courier New font subtending 0.3° by 0.4° of visual angle at a viewing distance of approximately 50 cm. In the AB task with grey dots distractors and in the AB task with red dot detection (described below), grey (40.2 cd/m^2^) dots with a diameter of 10 pixels were used. The red (28.8 cd/m^2^) dot used in the red dot detection task had the same size.

### Procedure

The experiment consisted of four practice blocks and three experimental blocks containing one of three different tasks: a standard AB task, an AB task with a concurrently moving irrelevant peripheral grey dot (grey dots task), or an AB task in which a short color change in the peripheral dot had to be detected (red dot task), as described below. The order of these blocks was counterbalanced between participants.

The first practice block always contained the standard AB task, and consisted of 108 trials. In each subsequent testing block, participants performed one of the three different tasks, preceded by a practice block of that specific task. One testing block (containing the red dot task) consisted of 288 trials whereas the other two blocks consisted of 216 trials. Each of the preceding practice blocks consisted of 12 trials. Between each block, the participants could take a short break. After half of the trials in each testing block, participants could take another short break.

The standard AB task required the identification of two letter targets amongst a rapid stream of digit distractors. Prior to each trial, a fixation cross was presented in the middle of the screen. Participants were instructed to fixate on the cross and to press the space bar to start the trial. After pressing the space bar, the fixation cross remained on the screen for a duration of 750 ms, followed by a blank screen. After 100 ms, a rapid serial visual presentation (RSVP) stream appeared in the middle of the screen, consisting of 22 sequentially presented stimuli. Each stimulus appeared for 90 ms without inter stimulus interval. In two-thirds of the trials, two targets were presented. In one-third of the trials, no targets were presented. In all target trials, the first target (T1) was presented as the fifth item in the stream, with the second target (T2) appearing as either the third (lag 3) or eighth (lag 8) item following T1. Known from the literature, an AB is likely to occur when T2 is presented at lag 3 (i.e. 270 ms after T1 onset), and unlikely to occur when presented at lag 8 (i.e. 720 ms after T1 onset).

The targets were randomly chosen from the set of target letters, with the only constraint that T1 and T2 were always different letters. The distractors were randomly chosen from the set of digits with the constraint that two successive digits were never identical. Participants were instructed to report the two targets at the end of the stream by pressing the corresponding letters on the keyboard. Participants were instructed to take sufficient time in making their responses to ensure that typing errors were not made. Whenever participants did not see a target, they were instructed to press the spacebar instead. Participants were encouraged to type in the letters in the order in which they had been presented, but responses were accepted and counted correct in either order. No feedback was given during the experiment.

The grey dots task was identical to the task described above, except that an irrelevant grey dot circled around the RSVP stream in synchrony with the presentation of each stimulus (i.e. the dot moved every ∼90 ms). The grey dot started randomly at one of 39 possible positions in a radius of 11.3° from the middle of the screen. The dot skipped two positions in clockwise rotation each time it moved. Participants were instructed to ignore the grey dot.

The red dot task was identical to the AB task with grey dots, except for the following changes. Participants were instructed to identify the targets in the AB task, while concurrently paying attention to the circling grey dot. In 25% of the trials, a red dot instead of a grey dot was presented for 90 ms, appearing once at a random time interval throughout the trial. After having responded to the target letters, participants were required to report whether or not they had seen the occurrence of a red dot (“press ‘j’ in case of a red dot, press ‘n’ in case of no red dot”). Importantly, only the trials during which no red dot appeared (75% of the trials) were considered in the analyses. Note that the perceptual input during these trials is identical to that in the AB task with irrelevant grey dots, with only the instructions being different. As mentioned above, the block of the red dot task contained more trials to keep the number of trials without a red dot occurrence equal to the number of trials in the blocks of the standard and grey dots tasks.

Although the order of the trials within each block was randomized, each condition (lag 3 or 8, targets or no targets, red dot present or absent) was balanced within a testing block. To reduce eye blink artifacts in the EEG recordings, participants were instructed to avoid making eye blinks during a trial until they were prompted to give their responses.

### EEG Recording

The EEG signal was recorded using a 64-channel electro-cap with tin electrodes. The electro-cap was organized according to the international 10/20 system and connected to an REFA 8–64 average reference amplifier. Impedance was reduced to less than 5kΩ for all electrodes. Data was sampled with a frequency of 2kHz and digitally reduced to 500Hz. Two electrodes connected to the mastoids served as an offline reference. The horizontal electrooculogram (EOG) was recorded from tin electrodes attached approximately 1 to 2 cm to the left and right of the outside corner of each eye. The vertical EOG was measured from two tin electrodes placed approximately 3 cm below the left eye and 1 cm above the brow of the left eye, respectively. Brain Vision Recorder (Brian Products GmbH, Munich, Germany) was used to control the data acquisition.

### Data Analysis

The preprocessing of the EEG data was done using Brain Vision Analyzer (Brain Products). The ERPs were time locked to the onset of the RSVP stream, had a duration of 2000 ms, and were calculated relative to a 200-ms prestream baseline, yielding a total length of 2200 ms. The ERP-segments were 40-Hz low-pass and 0.2-Hz high-pass filtered, and corrected for eye-movements. Segments with value differences greater than 100µV (i.e., containing artifacts) were excluded in the analysis. Artifact rejection excluded 5.7% of all trials (ranging from 0.19% to 25.6% per participant, SD = 6.4).

Both the performance and mean activity of the EEG data were analyzed using linear mixed effects models. The peak latencies were determined by peak detection over the averages per participant, therefore repeated measures ANOVAs were used to analyze peak latencies. The use of mixed effect models in the field of AB research is new, but the method is widely used in other fields (e.g., psycholinguistics [17], eye movement data [18] or memory research [19]). Given that there are large individual differences in the AB and that our hypothesis predicted a different number of observations per cell, it was inappropriate to analyze results using a method that assumes an equal number of observations per cell [17]. Therefore, mixed-effects models were used to analyze most datasets, but repeated measures ANOVAs were used to analyze the averaged latency data. Another advantage of mixed effect models is that these models are suited to analyze data from non-normal distributions, such as data based on accuracy scores [20]. For the ERP-based analyses, the default, normal distributions were used. For the behavioral data, a binominal model was used to fit the accuracy scores. The *p*-values reported for the non-binominal models of the EEG data were calculated by performing 10000 Markov Chain Monte Carlo (MCMC) samplings. Analyses were performed using the lmer and pvals.fnc functions in the lme4 and languageR packages [17], [21] for the statistical software R [22].

For the analysis of the behavioral data, two mixed-effects models were fitted. In the first model, both the grey dots and red dot tasks were compared to the standard task. Performance on lag 8 trials of the standard task was taken as reference in the first model. In order to investigate differences between the grey dots task and the red dot task directly, a separate mixed-effects model was fitted on the accuracy data with exclusion of the standard task. The grey dots task at lag 8 was taken as reference in this model. The standard task was excluded from all EEG-data analyses because of the differences in perceptual input induced by the standard task compared to the grey dots and red dot task. As the perceptual input of the grey dots and red dot task is identical, any differences found between the two tasks must be attributed to the manipulation of task instructions.

## Results and Discussion

### Behavioral Results

**Figure 1:**
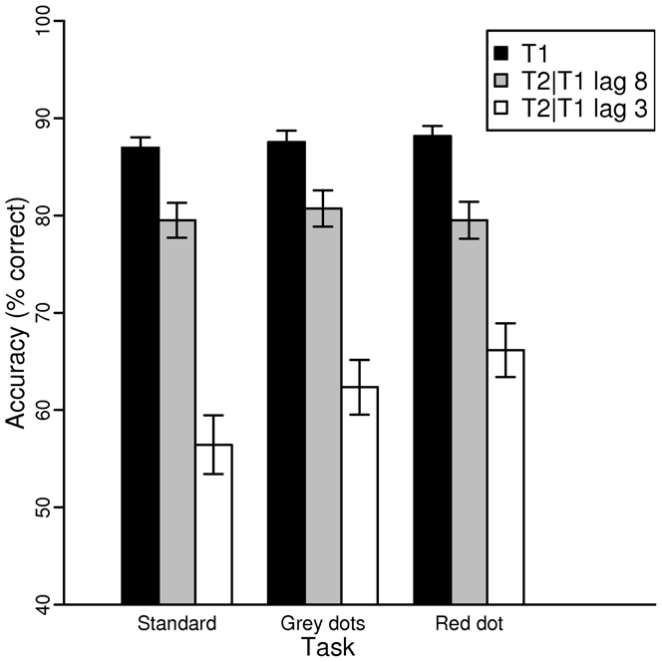
Accuracy scores of the standard, grey dots and red dot task for T2 given T1 is correct on lag 8 (dashed line) and on lag 3 (solid line).

Trials in the red dot task during which the red dot actually appeared were excluded from the analysis. On such trials, the red dot itself could possibly induce an AB on the first or second target. Detection accuracy for presence of the red dot was 65.1% (SD = 29.3). Figure 1 shows the identification performance on the AB tasks as a function of lag for T1 and T2 given that T1 was correctly reported (T2|T1).

#### T1 Accuracy

Two binominal mixed effects model were fitted on the accuracy of T1 with Task entered as fixed factor. Subject was entered as a random factor. The first model was fitted on all three tasks, using performance on the standard task at lag 8 as reference. In the second model, data of the standard task were left out in order to directly compare the grey dots task versus the red dot task. Table 1 lists the estimates and z-statistics of the first model. The estimates of the coefficients are reported in log odds. The coefficients of Task for the grey dots task and red dot task were not significant in the model, suggesting that both tasks did not affect overall performance. In the second model, the grey dots task was directly compared to the red dot task. In this model, also no significant effect of Task was found.

**Table 1:** The estimates and z-values of the mixed-effects model for T1 accuracy.

	Mixed-effects model T1			
	Estimate	Standard Error	z-value	p-value
Standard (Intercept)	2.111	0.167	12.643	0.000
Grey dots	0.056	0.075	0.749	0.454
Red dot	0.120	0.076	1.587	0.113

#### T2 Accuracy

In order to examine the effect of Task on the AB, a binominal mixed effect model was fitted on accuracy scores of T2 given correct report of T1. As in the analyses of T1, two models were fitted with Lag and Task entered as fixed factors, and Subject as random factor. As in the analysis of T1 accuracy, the data of the Standard task were left out of the analyses in the second model. The estimates and z-statistics of the first model are listed in Table 2. A main effect of Lag was found, such that performance was lower at lag 3 than at lag 8, reflecting the occurrence of an AB. No main effect of Task was found. However, significant Lag×Task interaction effects were found with a positive direction for the red dot task relative to performance on the standard task, reflecting that at lag 3 target performance was higher in the red dot task than in the standard task. No significant differences were found between the standard task and the grey dots task. A second model comparing the grey dots task and the red dot task directly reveals similar differences as found in the first model. Again, a main effect of Lag is found (*z* = −11.524, *p* = 0.000), indicating the presence of the AB effect. In addition, a significant Lag×Task interaction was found, (*z* = 2.047, *p* = 0.041), such that lag 3 performance was better in the red dot task than in the grey dots task. No main effect of task was found (*p* = 0.442). Taken together, a smaller AB effect was observed in the red dot condition compared to the standard AB task and the grey dots task. Although performance seemed better in the grey dots task than in the standard task (see Figure 1), no significant difference was found. The grey dots task thus seemed to be a suitable control task for comparison with the red dot task in the ERP analyses.

**Table 2:** The estimates and z-values of the mixed-effects model for T2|T1 accuracy.

	Mixed-effects model T2|T1			
	Estimate	Standard Error	z-value	p-value
Standard, Lag 8 (Intercept)	1.477	0.145	10.159	0.000
Grey dots	0.086	0.095	0.914	0.361
Red dot	0.017	0.094	0.186	0.852
Lag 3	−1.192	0.086	−13.791	0.000
Grey dots, Lag 3	0.184	0.123	1.496	0.135
Red dot, Lag 3	0.432	0.123	3.523	0.000

### Electrophysiological Results

As mentioned above, ERP amplitudes were analyzed using mixed effects models. As behavioral performance was similar in the standard AB and the grey dots tasks, we included only the grey dots task and the red dot task in the ERP analyses, as both task included the same perceptual input (as mentioned before, red-dot present trials were excluded from all analyses). Because the P3 was maximal at the Pz electrode, this electrode was used to analyze P3 activity. Based on visual inspection of the grand averages, a time window of 320–640 ms after target onset was used to determine P3 amplitude (in terms of mean activity). For each individual and condition, the latencies of the P3 peaks were obtained by searching for the maximum peak in the before-mentioned time window per condition-based average. To determine the FSP amplitudes, target- and distractor-related activity was analyzed in a window of 180–340 ms after the onset of T1. Target-related activity was obtained by taking activity within that window on trials during which T1 was successfully reported, whereas distractor-related activity was obtained by taking the activity of the non-target trials within the same time window.

#### The P3 On Lag 8 Trials

**Figure 2:**
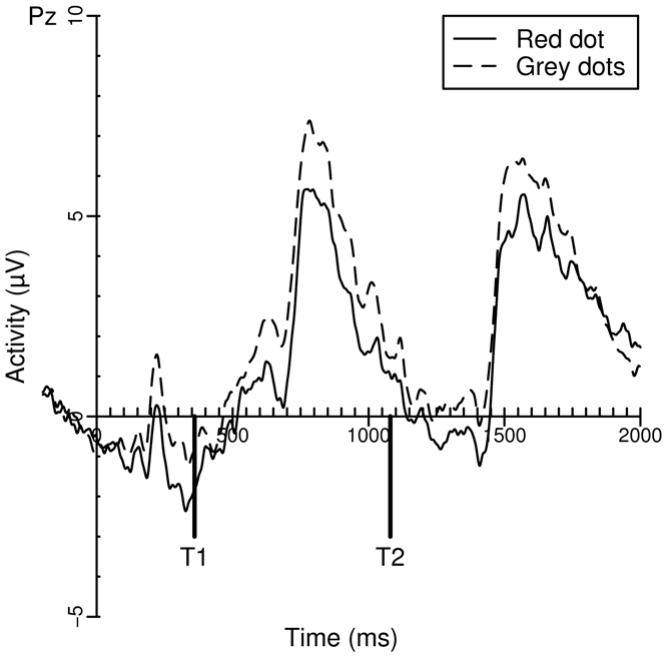
Grand averages of Pz on lag 8 correct trials for the grey dots (dashed line) and red dot (solid line) task.

Figure 2 shows the grand averages of activity at Pz during lag 8 trials, given that T1 and T2 were both correctly identified. The P3 activity of both T1 and T2 was analyzed using a mixed effect model. Task (Grey dots or Red dot) and Target (T1 or T2) were entered in the model as fixed factors. As in the analyses of the behavioral data, Subject was entered as a random factor. The estimates of the model are listed in Table 3. In the model, there was a significant effect of the red dot task. Whenever participants were engaged in the red dot task, P3 mean activity was lower than in the grey dots task. To determine whether the decrement in activity was specific for the P3 component, a second analysis was performed with a baseline from −200 to the onset of the P3 component (i.e. 680 ms). Again, there was a significant difference in P3 amplitude between the grey dots task and the red dot task (*t* = −3.386, *p* = 0.000), suggesting that there was indeed a target-specific decrement in activity on top of a possible overall reduction in activity. A separate repeated measures ANOVA on the P3 peak latencies of the first target with Task as within-subjects factor did not reveal any significant difference between the tasks (*F*<1).

**Table 3:** The estimates and t-values of the mixed-effects model for P3 amplitude (measured in mean activity) at lag 8 trials.

	Mixed-effects model			
	Estimate	Standard Error	t-value	p-value
Grey dots, T1 (Intercept)	5.037	0.653	7.709	0.000
Red dot	−1.455	0.353	−4.120	0.000
T2	−0.284	0.346	−0.821	0.410
Red dot, T2	0.081	0.499	0.162	0.870

#### The P3 On Lag 3 Trials

**Figure 3:**
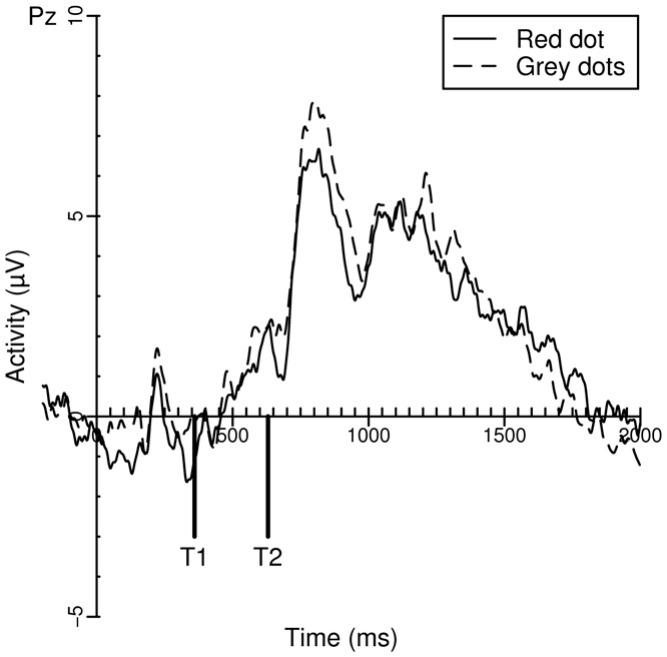
Grand averages of Pz on lag 3 correct trials for the grey dots (dashed line) and red dot (solid line) task.

The grand averages of Pz for lag 3 trials can be found in Figure 3. To investigate the effect of task on the amplitude P3 of T1 and T2, all lag 3 trials in which T1 was correctly reported were analyzed using a mixed-effects model. Target (T1 or T2) and Task were entered as fixed factors. Subject was entered as a random factor. The estimates and t-values of the coefficients of both models can be found in Table 4. A significant effect of Task was found, such that the P3 was smaller in the red dot task than in the grey dots task. Neither a significant difference was found for Target nor for the interaction between Task and Target. Again, a second analysis was performed with a baseline from −200 to 680 ms to see whether an analysis corrected for a possible overall negativity would yield similar results. A significant effect of the red dot task on P3 amplitude was obtained (*t* = −2.205, *p* = 0.027), suggesting that the target-specific decrement in activity found on lag 8 trials was also present on lag 3 trials. A repeated measures ANOVA of the P3 latencies with Task as within-factor did not reveal any significant main effects or interactions (*F*<1).

**Table 4:** The estimates and t-values of the mixed-effects model for P3 amplitude (measured in mean activity) at lag 3 trials.

	Mixed-effects model			
	Estimate	Standard Error	t-value	p-value
Grey dots, T1 (Intercept)	5.336	0.760	7.028	0.000
Red dot	−0.933	0.375	−2.492	0.013
T2	−0.475	0.376	−1.264	0.205
Red dot, T2	0.4286	0.529	0.810	0.416

#### Distractor-related Activity

**Figure 4:**
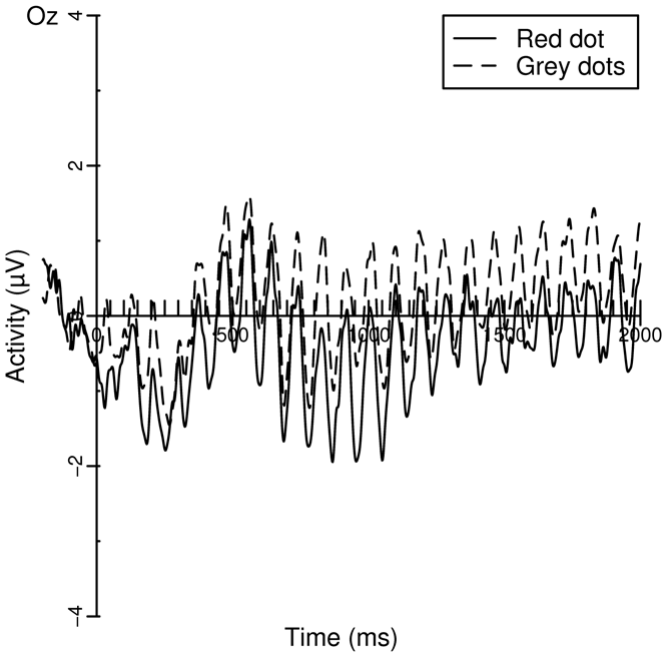
Grand averages of activation on Oz for the grey dots (dashed line) and red dot (solid line) task.

To investigate distractor-related activity, mean activity on no-target trials was analyzed using mixed effect models. Distractor-related activity differed the most at the Oz electrode. Figure 4 shows the mean activity at Oz on the no-target trials for all three tasks. A mixed-effects model with Task entered as fixed factor and Subject entered as Random factor revealed a significant effect of the red dot task. As shown in Table 5, mean activity was significantly lower in the red dot task when compared to the grey dots task.

**Table 5:** The estimates and z-values of the mixed-effects model for Oz activity when no targets are presented.

	Mixed-effects model A			
	Estimate	Standard Error	t-value	p-value
Grey dots (Intercept)	0.217	0.514	0.421	0.692
Red dot	−0.724	0.216	−3.354	0.001

#### The FSP

Activity on F7 and F8 electrodes was analyzed using a mixed effect model. Task, Hemisphere and Targets (where in this case Targets coded for either the presence or absence of a target) were entered in the model as fixed factors. Subject was entered as random factor. A significant effect was found for Targets (*t* = 3.328, *p* = 0.002), indicating the presence of a significant FSP in all three tasks. No other main effects or interactions were found to be significant (*p*s>0.17). To determine peak latency, the latency of the maximum positive peak within the time window of 180–340 ms after target onset was taken. A repeated measures ANOVA with Task as within-subjects factor did not reveal a significant difference in peak latency (*p*>0.32)

### General Discussion

Previous research has shown that distracting participants during an AB task improves performance in identifying T2 during the blink period [3]–[6]. In the current study, behavioral as well as electrophysiological effects of a red dot detection task on a concurrent AB task were investigated. Performance in this dual task setting (referred to as the red dot task) was compared to that in a single AB task (standard task). As a second control condition, a task with similar perceptual input as in the red dot task was also included in the experiment (grey dots task). In contrast to the red dot detection task, the dots that appeared in the latter task were simply to be ignored.

Compared to both control tasks, participants showed an attenuated AB in the red dot task. This result is in line with previous findings reported by Taatgen [6] and colleagues, despite various small methodological differences between the experiments (i.e., in counterbalancing the order of conditions, the presence of feedback, and the number of lags, distractors, and targets). Most importantly, we found evidence that the P3 amplitudes induced by successfully identified targets were reduced during both lag 3 and lag 8 trials of the red dot task. The finding that P3 amplitude decreases when engaged in a second task is in line with these results [16].

The finding of reduced P3 amplitudes corresponds nicely with previous reports of a relationship between P3 amplitude and AB magnitude [13], [14]. Whereas Martens et al. manipulated P3 amplitude using cuing and stimulus probability, McArthur et al. changed the response set size of T1. Both research groups found that a larger P3 coincided with a larger AB magnitude. More evidence is provided by a MEG study, in which a positive correlation was found between an individual's P3 (or M300) amplitude and AB magnitude [15].

Martens et al. [12] specifically focused on individual differences in AB magnitude by studying a group of so-called ‘non-blinkers’, who show little or no AB, and a group of strong ‘blinkers’, who show a large AB magnitude. Compared to blinkers, non-blinkers showed more target-related frontal activity (reflected in the FSP component), less distractor-related frontal activity (on target-absent trials), and earlier peak latencies of the target-induced P3s. Martens and colleagues argued that non-blinkers consolidate relevant information quicker (evidenced by the earlier P3 peak latencies), presumably because they are capable of making an early selection, discriminating targets from distractors at an early stage of processing (reflected in increased target-related and decreased distractor-related frontal activity).

An important question that we wanted to address in the current study was whether distraction during the AB task turns blinkers into non-blinkers. The answer to that question seems to be a tentative “no”. Firstly, although there was a significant reduction in AB magnitude in the red dot task, participants in the current study still showed a much larger AB than the non-blinkers did in previous studies [8], [12], [23]. Although, like the non-blinkers, participants in the red dot detection task showed a significant reduction in distractor-related activity, this reduction was most prominent over occipital rather than frontal brain areas. In addition, neither a change in P3 peak latency nor in FSP amplitude was observed in the current study. These findings may thus be taken to suggest that ‘natural’ non-blinkers and the ‘induced non-blinkers’ from the current study differ fundamentally in the way they perform the AB task.

As suggested in a recent study by Martens, Korucuoglu, Smid, and Nieuwenstein [24], natural non-blinkers might be more efficient than blinkers to take advantage of overlearned category-level features to select targets prior to full identification, allowing natural non-blinkers to *ignore* rather than *suppress* distractors, thereby avoiding an AB (also see Taatgen et al. [6]).

The picture that tentatively emerges is that the amplitude of the P3 induced by T1 plays an important role in determining whether an AB occurs. Polich [25] argued that P3 amplitude is related to inhibition processes occurring in the brain. The function of this inhibition process is speculated to protect the target stimulus from interference by competing stimuli. Given the existence of non-blinkers and supported by computer simulations, Taatgen et al. [6] hypothesized that this inhibition/protection is in fact unnecessary. Any reduction in P3 amplitude (or shift in latency) may imply that less inhibition is exerted on the items following T1, including T2, thereby increasing the chance of successful T2 report. Not only is this in line with what we observed in the red dot task, this view is also consistent with many theories that attribute the AB to an inhibition process that is induced while the first target is being consolidated (e.g., [6], [10], [26]–[28]).

In addition to a reduction of target-related parietal activity, we also found a reduction of distractor-related occipital activity. Research on sensory-evoked responses in early visuocortical processing has shown that increased perceptual load results in smaller P1 amplitudes (a component associated with early attention processes [29]) at the occipital sites, accompanied by a reduction of distractor interference on target processing [30], [31].

To summarize, we found that the AB is attenuated by the addition of a secondary task. In addition, a target-specific reduction in P3 amplitude was found on top of a general decrement in overall distractor-related activity. The presence of a secondary task in the present study presumably increased the perceptual/cognitive load, thus leading to reduced distractor processing (reflected in the reduced distractor-related occipital activity). We conclude that this reduction in distractor processing in turn leads to a reduced ‘need’ for inhibitory processes later in the processing pathway (reflected in reduced P3s), and therefore a reduced AB. The present findings bring us a step closer in understanding why and how an AB occurs, and how these temporal restrictions in selective attention can be overcome.
